# Site characteristics determine the effectiveness of tillage and cover crops on the net ecosystem carbon balance in California vineyard agroecosystems

**DOI:** 10.3389/fpls.2022.1024606

**Published:** 2022-11-21

**Authors:** Maria Zumkeller, Runze Yu, Nazareth Torres, Lauren E. Marigliano, Daniele Zaccaria, Sahap Kaan Kurtural

**Affiliations:** ^1^ Department of Viticulture and Enology, University of California, Davis, Davis, CA, United States; ^2^ Department of Land, Air and Water Resources, University of California, Davis, Davis, CA, United States

**Keywords:** climate change, carbon sequestration, cover crops, tillage activities, soil conservation

## Abstract

Globally, wine grape vineyards cover approximately 7.4 M ha. The potential for carbon (C) storage in vineyards is of great interest to offset greenhouse gas emissions and mitigate the effects of climate change. Sustainable soil management practices such as cover crop adoption and reduced tillage may contribute to soil organic carbon (SOC) sequestration. However, site-specific factors such as soil texture, other soil physicochemical properties, and climate largely influence the range and rate to which SOC may be stored. To measure the potential for C storage in vineyards under varying sustainable soil management practices, we calculated the net ecosystem carbon balance (NECB) of three cover crops [perennial grass (*Poa bulbosa* hybrid cv. Oakville Blue); annual grass (barley, *Hordeum vulgare*); resident vegetation (natural weed population)] under conventional tillage (CT) and no-till (NT) management. Results provided evidence that vineyards served as C sinks. In sandy soils, the type of cover crop and tillage may be of little influence on the NECB. While in finer-textured soils, tillage reduced the NECB and higher biomass-producing cover crops enhanced the overall C storage potential of the vineyard agroecosystem. Overall, our results revealed that site characteristics, namely, soil texture and climate, were key determinants of the C storage potential of vineyards in Mediterranean climates such as those found in coastal and inland California wine grape production regions.

## 1 Introduction

As temperatures rise and rain events become more unpredictable due to the changing climate, soils are under threat of loss of soil organic matter (SOM), soil nutrient imbalances, loss of soil biodiversity, contamination, and compaction (Panagos et al., 2020). Moreover, it is estimated that almost 36 billion tons of soils are lost annually due to water and wind erosion (Alsina et al., 2014; Borrelli et al., 2017; Wolff et al., 2018). Soil erosion is further exacerbated by tillage, and an overreliance on soil cultivation for weed control and aeration over the past half century has resulted in a significant loss of SOM across agricultural soils (Alsina et al., 2014; Mitchell et al., 2017). Thus, over the last decade, there has been a substantial increase in attention toward rebuilding SOM and using soils as a tool to mitigate climate change (Lal, 2004; Powlson et al., 2011; Lazcano et al., 2020; Nilahyane et al., 2020).

**Figure 1:**
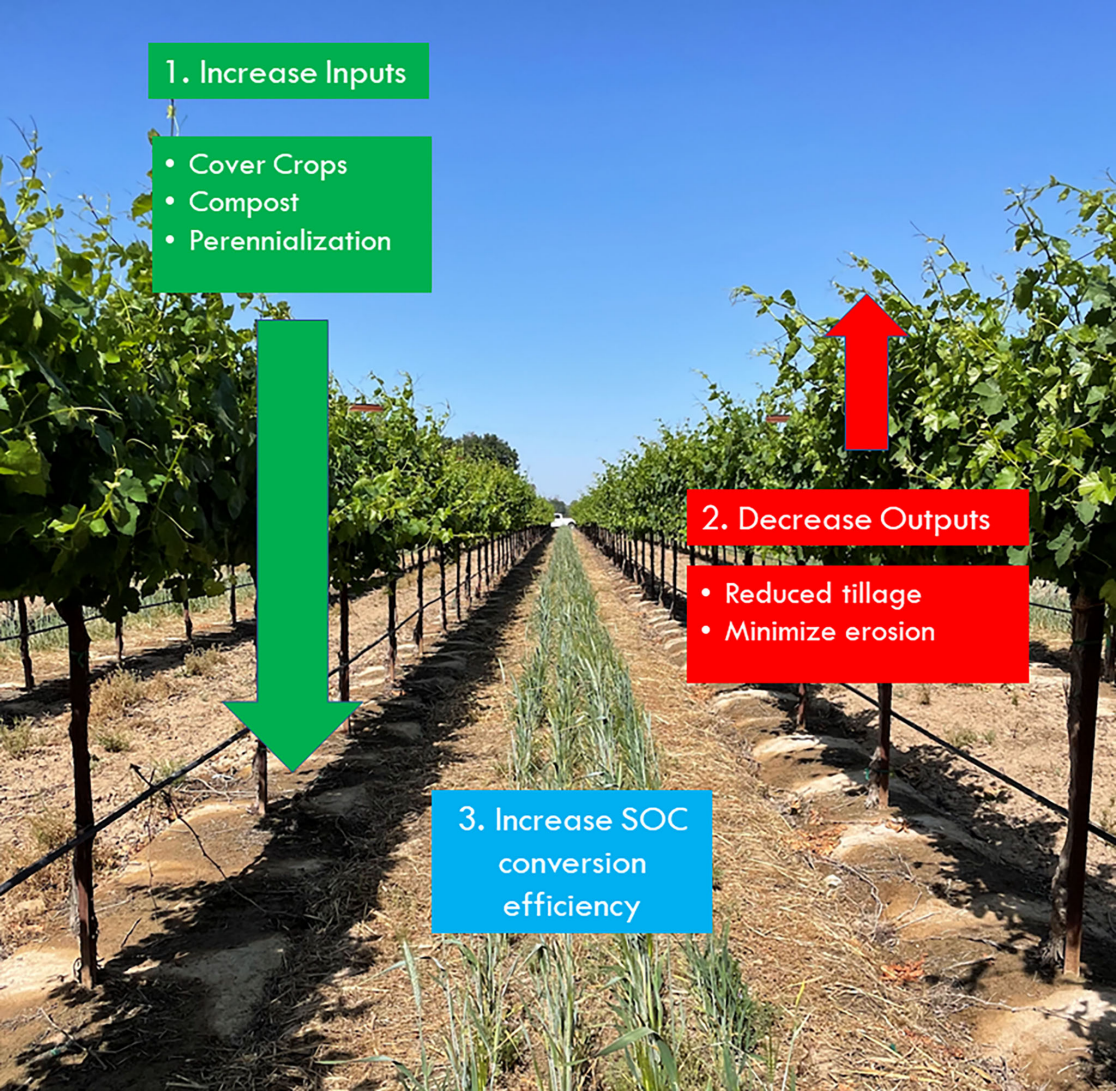
Framework for increasing soil organic carbon (SOC) conversion effiency in vineyards by increasing inputs of cover crops, compost, and perennializaiton and decreasing outputs from tillage and erosion avoidance.

Traditionally, the interrows of vineyards were kept free of vegetation with the use of herbicides and tillage. However, it has been shown that both practices may have detrimental effects on soil quality and the surrounding ecosystem (Patiño-Zúñiga et al., 2009; Ferreira et al., 2020; Gatti et al., 2022). Thus, the adoption of cover crops and reduction of interrow tillage have been proposed as sustainable alternatives to conventional vineyard floor management practices (**Figure 1**) (Alsina et al., 2013). Research suggested that cover crops may not only reduce soil erosion and water runoff but also improve water infiltration in most soils of temperate regions by increasing SOM, so soils and water can be better conserved (Álvaro-Fuentes et al., 2008; Steenwerth and Belina, 2008a; Belmonte et al., 2018; Cataldo et al., 2020). In addition, SOM can be further preserved under reduced till or no-till (NT) practices, whereby soil aggregates and accompanying SOM remain undisturbed (Šimon et al., 2009; Peregrina et al., 2010; Seddaiu et al., 2013). The preservation of SOM in turn bolsters soil organic carbon (SOC), which would ameliorate the soil physical, chemical, and biological functions and was identified as a key target carbon (C) pool in mitigating climate change *via* C sequestration (Jobbágy and Jackson, 2000; Blanco-Canqui et al., 2013; Stockmann et al., 2013). In fact, the Intergovernmental Panel on Climate Change (IPCC) has estimated that by 2030, global SOC sequestration has the potential to mitigate up to about 5.3 Gt CO_2_ per year (Porter et al., 2017). Vineyard agroecosystems represent a large potential for agricultural soil carbon sequestration (SCS): grape vineyards, including wine, table, and raisin grapes, make up 341,555 hectares (844,000 acres) of agricultural land in California [California Department of Food and Agriculture (CDFA), 2022]. Also, the large SCS potential can attribute to the grapevine’s long life cycle and permanent woody organs allow them to potentially store higher amounts of C compared to annual crops (Alonso et al., 2014; Nistor et al., 2018). However, there are limitations to SCS, including a lack of standardized methods of SOC determination and uncertainty regarding the stability of different soil C pools (Powlson et al., 2011). Furthermore, the effectiveness and rate of long-term SOC sequestration in agricultural soils can be largely influenced by site-specific conditions including climate, soil texture, other soil physiochemical properties, and management practices (Carlisle et al., 2010; Powlson et al., 2011).

Previous studies have indeed identified vineyards as C sinks. However, the management practices largely influenced the storage potential of the vineyard systems (Brunori et al., 2016; Payen et al., 2021). Subsequently, management practices that increase SOM have been encouraged over the last decade as an SCS strategy, yet their impact on SOC storage rates remains unclear (Novara et al., 2019). While some studies have recorded an increase in SOC sequestration rate due to cover crop adoption (Steenwerth and Belina, 2008a; Alonso et al., 2014), others have reported the opposite (Celette, 2007; Novara et al., 2019; Jian et al., 2020). Also, the effectiveness has shown significant connections with the longevity of the management practices and site characteristics (Krull et al., 2001; Mitchell et al., 2017).

One source of limitation in quantifying C sequestration in previous literature may be the presence or absence of measurement of CO_2_ efflux from assessing soil respiration (R_s_). Soil CO_2_ efflux results from the combination of biological and physical processes, both of which are sensitive to edaphic factors and highly variable in space and time (Ryan and Law, 2005). In soils of hot climates, CO_2_ efflux may be great enough to potentially offset short-term storage of C from cover crop adoption (Yu et al., 2019). Furthermore, some studies have shown that cover crops enhance microbial activity, thus increasing CO_2_ efflux (Steenwerth et al., 2010; Maier et al., 2011; Freidenreich et al., 2021). In fact, CO_2_ emissions may be further increased under specific types of cover crops due to the lower C:N ratios of certain plant materials, such as legumes (Alluvione et al., 2010; Freidenreich et al., 2021). Since recent work has shown that SOC increases as tillage frequency or intensity is reduced, some studies have investigated the synergic effects of cover crops combined with reduced till or NT management in California vineyards (Conant et al., 2007; Steenwerth and Belina, 2008a; Wolff et al., 2018). However, there is still a lack of information regarding the potential of C storage in vineyard systems under combinations of soil management practices, considering C stored or lost at vineyard scale.

Thus, a net ecosystem carbon balance (NECB) is needed to elucidate C inputs and outputs in commercial production settings at vineyard scale. One method of estimating net C storage within a system is through the net ecosystem production (NEP) methodology (Randerson et al., 2002). The NEP reflects the balance of ecosystem primary production as stored C minus ecosystem respiration as lost C to determine whether there is net gain or loss of C in the monitored ecosystem. On the other hand, the NEP is effectively parallel to the net primary production (NPP), as NPP only quantifies the C input by plants, but NEP includes the consideration of C input by plants in addition to C release by soils. Therefore, by combining the NPP of both cover crop and grapevine with soil CO_2_ efflux and C losses as grape clusters and canes being removed during harvest and dormant season, respectively, the NECB can be quantified to reveal the C gain or loss at vineyard scale (Cates and Jackson, 2019).

Thus, the objective of this study was to investigate the synergic effects of the implementation of cover crops and NT practices by quantifying C inputs and losses at the vineyard scale through the NECB determination in two different wine production regions and to investigate the contributions of specific site characteristics toward these vineyard floor management practices in two hyperarid seasons in California. We hypothesized that the vineyard agroecosystem can serve as a C storage pool, and the effectiveness of the ecological functioning of NT and cover crops will be determined by site characteristics, including climate and soil texture.

## 2 Materials and methods

### 2.1 Experimental design

Field experiments were conducted at two sites for two consecutive growing seasons (2019–2020 and 2020–2021). The first site was located in Five Points, Fresno County, CA, USA (36.671514, -119.925823), in a Ruby Cabernet/Freedom (27% *Vitis vinifera* hybrid) vineyard. Grapevines were planted in 2012 with a spacing of 3.0 m × 1.2 m (row × vine) with a row orientation of east to west (E–W). The grapevines were head trained and cane pruned. The vineyard was trained to a high quadrilateral trellis with fruiting wire at 1.54 m and catch wires at 1.68 m above the vineyard floor. The grapevines were drip irrigated with two emitters per plant delivering 4.0 L/h each. The second site was located in Oakville, Napa County, CA, USA (38.428, 122.409), planted with Merlot (clone 181)/3309 C (*Vitis riparia* × *Vitis rupestris*). Grapevines were planted in 2018 with a spacing of 3.0 m × 2.0 m (row × vine) with a row orientation of E–W. The grapevines were spur pruned and trained to quadrilateral cordons 1.54 m above the vineyard floor with catch wires at 1.68 m. The grapevines were drip irrigated with two emitters per plant delivering 2 L/h each.

At both sites, experiments were arranged in a split-plot 3 × 2 factorial design (three different cover crops subjected to two tillage managements) with four (Napa) and three replications (Fresno). At both vineyards, each treatment replicate consisted of 15 grapevines. Three vines in the middle of each replicate were used for on-site measurements, including the parameters from both grapevines and soils under grapevines, while the distal vines on either end were treated as border plants. Cover crop treatments included 1) perennial grass (PG) (*Poa bulbosa* hybrid cv. Oakville Blue), 2) annual grass (AG) (barley, *Hordeum vulgare*), and 3) resident vegetation (RV) (natural weed population). Tillage management consisted of NT in which interrows were disked 2–3 cm only once in the fall as preparing for seeding and conventional tillage (CT) that was disked to a depth of 10 cm once in the fall and twice in the spring to incorporate the cover crop residue. Tillage events in Fresno occurred on 1 November, 16 April, and 25 June for 2020 and 12 October, 28 April, and 18 June for 2021. Tillage events in Oakville occurred on 9 October, 21 April, and 24 June for the 2020 season and 2 October, 16 April, and 17 June for 2021. The cover crop seed was drilled to a 1.5-m-wide strip according to seed manufacturer’s recommended practices prior to receiving fall/winter rains in 2019 and 2020 at a rate of 605 kg/ha and 84 kg/ha for the PG and AG treatments, respectively. RV was allowed to grow within a 1.5-m strip in the interrow and mowed according to vineyard manager’s discretion. Berms were 1.0 m wide and kept free of vegetation using a glyphosate herbicide application in the spring.

### 2.2 Site conditions

The site conditions in both Fresno County and Napa County vineyards are presented in **Table 1**. Annual mean daily air and soil temperatures, as well as maximum and minimum air temperatures (1 January to 31 December), from the two sites were obtained from the California Irrigation Management Information System (CIMIS) stations nearest the experimental vineyard (station #77 in Napa County, CA, and station #2 in Fresno County, CA, USA).

**Table 1:** Site conditions at two commercial vineyards in Fresno County and Napa County from experimental years (2019–2021) and long-term mean values (2011–2021).

Year	Air temperature (°C)			Soil temperature (°C)			Precipitation (mm)^b^	GDD (°C)
	Daily max	Daily min	Daily average	Daily max	Daily min	Daily average		
**Fresno County (Five points)**								
**2020**								
Mean	25.6	9.1	17.3	25.7	10.7	22.6	199	2,358
Annual max^a^	35.8	18	27.3	--	--	--	--	--
Annual min^a^	15.6	0.6	7.6	--	--	--	--	--
**2021**								
Mean	25.8	9.2	17.6	26.6	10.4	22.8	152.5	2,488
Annual max	37.8	18.7	28.5	--	--	--	--	--
Annual min	12.5	3.1	7.7	--	--	--	--	--
**2011**–**2021**								
Mean	25.4	9.4	17.2	25.6	10.1	22.3	209.6	2,259
**Napa County (Oakville)**								
**2020**								
Mean	24.4	7	14.9	22.5	10	16.5	234.2	1,647
Annual max	31.8	12.3	21.1	--	--	--	--	--
Annual min	17.1	2	8.5	--	--	--	--	--
**2021**								
Mean	23.1	6.3	14.2	22.8	10.2	16.4	278.3	1,519
Annual max	30	10.8	19.2	--	--	--	--	--
Annual min	12.5	2.6	7.6	--	--	--	--	--
**2011**–**2021**								
Mean	23.2	7.1	14.5	22.8	7.9	16	577.8	1,504

^a^ Annual maximum (max) and annual minimum (min) indicate the greatest or lowest value observed during the respective year. ^b^ Total precipitation occurred during the annual winter rainy season, calculated from October of the preceding year through September of the following year (e.g., 2020 values were calculated from 1 October 2019 to 30 September 2020). ^c^--, not applicable and GDD, growing degree days.

At both sites, air temperatures were consistent between years. However, the mean daily maximum temperature was 0.2°C higher in 2021 than 2020, while it was 1.3°C lower in 2021 compared to 2020 in Napa County. Daily maximum soil temperature was 0.9°C and 0.3°C higher in 2021 compared to 2020 in both Fresno County and Napa County, respectively. Daily average soil temperatures were also slightly higher by a degree of 0.1°C–0.2°C at both sites in 2021. Furthermore, average soil temperatures during both years were 0.3°C–0.5°C higher in Fresno County and 0.4°C–0.5°C higher in Napa County compared to the long-term average for the region over the past 10 years (2011–2021) (**Table 1**).

At the Fresno County vineyard, approximately 199.0 mm of rain was received at the experimental site beginning in October of the preceding year until the harvest in year 1, while 152.5 mm of rain was received during the same period in year 2. In year 1 of the study, the greatest amount of precipitation was received in March (67 mm), while in year 2, the greatest amount (87 mm) was received in January followed by December and October. Therefore, compared to year 1, year 2 received more fall-to-winter precipitation. The Napa County vineyard received a greater amount of rainfall compared to that of Fresno County, as 234.2 mm of rain was received in year 1 and 278.3 mm of rain in year 2. December and January received the greatest amount of precipitation in both year 1 and year 2.

Soil texture was assessed using the hydrometer method (S-14.10) from the North American Proficiency Testing (NAPT) program. The SOC content was measured in the interrows of each experimental unit under dry conditions. In July 2020, bulk density was assessed at the centers of interrows (122 cm from the vine rows) and the edges of the berms (61 cm from the grapevine trunk) using brass rings of 10 cm internal diameter and 7.5 cm length. No differences in bulk densities were found in the soil samples between CT and NT interrows, thus all soil samples were taken at the same depth of 30 cm. Three soil cores were randomly collected per experimental unit to a depth of 30 cm and partitioned into 0–15 cm and 15–30 cm subsamples. Subsamples were homogenized and kept in a cool environment until analysis. At analysis, samples were dried, sieved to <2 mm, ball-milled, and analyzed for SOC by combustion method (S-9.30), and soil texture was determined by hydrometer analysis (S-14.10) according to the NAPT program. Soil pH was determined *via* the saturated paste method as described by Gavlak et al. (1994).

### 2.3 Soil respiration

To calculate the losses of soil C through R_s_, soil CO_2_ efflux was measured *in situ* using a CIRAS-3 (PP Systems, Amesbury, MA, USA) portable gas exchange system coupled with a closed system soil respiration chamber (SRC-2). The SRC-2 chamber consisted of a soil surface area of 78 cm^2^ and a system volume of 1,171 ml and was placed on the vineyard soil surface in the interrows and on the bare soil under the grapevines in each experimental unit to measure R_s_ at both locations. To minimize leakage, the chamber was fit onto a 10-cm-diameter polyvinyl chloride (PVC ring) placed 5 cm deep in the vineyard interrow and on the bare soil under the grapevines in each experimental unit. The PVC rings remained in the soil throughout the experiment except for removal for mowing and tillage whereby the ring was replaced at least 24 h prior to sampling. Sampling areas were selected with careful consideration for the least plant material to avoid CO_2_ contributions from aboveground plant parts. Additionally, as autotrophic and heterotrophic respiration could not be separated in this study, the measured CO_2_ efflux included emissions from all soil processes. Upon measurement, the chamber was allowed to stabilize for 1 min before the gas accumulated in the chamber headspace was continuously sampled in the closed circuit. Efflux rate of CO_2_ (µmol CO_2_ m^−2^ s^−1^) was calculated based on linear fit by the CIRAS-3 analyzer as shown in Eq. 1:

Eq. 1. $R_{s}=\frac{Cn-Co}{Tn}\times \frac{V}{A}$

where R_s_ is the respiration rate (CO_2_ flux, or moles of CO_2_ unit area^-1^ unit time^-1^), C_o_ is the CO_2_ concentration at T = 0, and C_n_ is the concentration at a time T_n_ later. A is the area of soil surface exposed (78 cm^2^), and V is the total system volume (1,171 ml). The air within the SRC-2 chamber was continuously and automatically mixed during the measurement period to ensure representative samples.

R_s_ measurements took place no more than 2 h after solar noon and was measured at six time points per experimental unit in each season in Oakville (24 January, 13 April, 21 April, 23 April, 22 May, and 19 June in 2020; 29 January, 14 April, 16 April, 14 May, 15 June, and 9 July in 2021) and five time points in Fresno (2 March, 25 March, 16 April, 17 April, and 12 June in 2020; 14 February, 24 March, 28 April, 29 April, and 1 July in 2021). Measurement time points were selected to represent soil conditions throughout the season, including the day before and after a tillage event and important precipitation events at both sites. Mean R_s_ values for the season were calculated for each treatment and the bare soil control. In the first year of the study, soil moisture was measured as the volumetric water content (VWC) at the time of each R_s_ measurement. No significant differences were found between treatments, and thus, soil moisture was not monitored the following season, and measurement dates were targeted before and after tillage and precipitation events.

### 2.4 Estimates of net primary productivity

#### 2.4.1 Grapevine net primary production

NPP_grapevine_ was estimated as the summation of annual production (harvest yield, leaf biomass, and cane production) and permanent organs (trunk and root biomass). Harvest commenced when the fruit reached approximately 25°Bx in Oakville (25 August 2020 and 1 September 2021) and 21°Bx in Fresno (6 October 2020 and 7 September 2021). At both sites, clusters from three data vines per experimental unit were manually removed, counted, and weighed on a top-loading balance. Subsamples were collected from clusters within each experimental unit at harvest, and C content (% mass) was determined *via* combustion (Western Region Method S-9.30) (Gavlak et al., 1994).

To assess the leaf biomass at the Fresno vineyard, leaf area index (LAI) was measured in late spring to characterize the grapevine canopy growth by a smartphone program, VitiCanopy, *via* iOS system (Apple Inc., Cupertino, CA, USA) (De Bei et al., 2016) and converted into leaf area based on the ground area (3.6 m^2^). The gap fraction threshold was set to 0.75, extinction coefficient was set to 0.7, and subdivisions were 25. An extendable mounting device was used to effectively position the device approximately 75 cm underneath the canopy. The device was positioned with the maximum length of the screen being perpendicular to the cordon, and the cordon in line with the middle of the screen according to previous work (De Bei et al., 2016; Yu and Kurtural, 2020). In each experimental unit, three images were taken to capture half canopy of each vine and analyzed by the software. Subsamples of 100 leaves were collected per experimental unit, and leaf area (cm^2^) was determined by leaf area meter (Li-Cor 3300, Lincoln, NE, USA), dried at 80°C. Dry weight (g) was recorded, and values were extrapolated to determine LAI as previously reported (Torres et al., 2021). At the Oakville vineyard, two vines per treatment were completely defoliated and biomass was measured. Carbon content (% mass) of leaves was estimated as 56% of dry weight (Zhang et al., 2021).

Cane production (pruning wood weights) was measured at dormancy among the three data vines per experimental unit. The C content (%) of pruning wood was estimated based on previous literature, which was 9% as the average percentage fractions of biomass of canes (Morandé et al., 2017). Annual biomass accumulation in permanent organs (trunk, cordon, and roots) was also acquired from literature and included one prior study based at the same experimental site (Oakville, CA) and another at a vineyard of similar age and productivity in the San Joaquin Valley (Williams et al., 2011; Martínez-Lüscher and Kurtural, 2021).

#### 2.4.2 Cover crop NPP

NPP_cover crop_ was calculated by collecting aboveground and belowground biomass at crop physiological maturity as described previously (Steenwerth and Belina, 2008b) or just before termination. A one, 1 m^2^ quadrat was randomly placed in the interrow of each experimental unit and all aboveground biomass was collected. Belowground biomass (roots) was also collected to a depth of 30 cm. Cover crop fresh biomass was determined and then dried at 60°C for 48 h to obtain the dry biomass. The C contribution (%) of the cover crop was estimated as 50% C of the dry biomass (Cates and Jackson, 2019).

### 2.5 Determination of the net ecosystem carbon balance

The NECB (Mg C ha^-1^ year^-1^) was calculated as follows:

Eq. 2. NECB = NPP_grapevine_ + SOC + NPP_cover crop_ – R_s: interrow_ – harvest – R_s: under vine_

where NPP_grapevine_ is the summation of annual (leaves and fruits) and perennial (permanent organs) growth, SOC is the soil organic carbon sequestered to a depth of 30 cm adjusted for the interrow spatial coverage, NPP_cover crop_ is the sum of aboveground and belowground cover crop biomass to 10 cm, R_s: interrow_ is the soil respiration of the portion of the vineyard where the cover crop is grown, harvest is the amount of C removed through yield, and R_s: under vine_ is the soil respiration of the portion of the soil left bare. Interrow coverage was estimated as 48% of one hectare and bare soil 52% of one hectare. A positive NECB signifies that the system is the net sink of C, and a negative NECB signifies a net source of C to the atmosphere.

### 2.6 Statistical analyses

Statistical analyses were conducted with R studio version 3.6.1 (RStudio: Integrated Development for R., Boston, MA, USA) for Mac OS. After normality assessment, data were submitted to a two-way analysis of variance (ANOVA) to assess the statistical differences between the different cover crop and tillage treatments and the respective interaction effects. Means ± standard errors (SEs) were calculated, and when the F value was significant (P ≤ 0.05), a Tukey’s “honest significant difference” (HSD) *post-hoc* test was executed by using “agricolae” 1.2-8 R package. Figures were made using GraphPad Prism v8.1.2 for Windows (GraphPad Inc., San Diego, CA, USA).

## 3 Results

### 3.1 Soil properties

The soil texture at the Fresno County vineyard is classified as a sandy loam with approximately 66% sand, 22% silt, and 12% clay and a bulk density value 1.4 g cm ^-3^. The SOC (% mass C) was not affected by type of cover crop nor tillage system over the course of the experiment at either depth in the Fresno County vineyard (**Table 2**). Although SOC was not significantly different between years at this site, greater SOC was observed in the upper 0–15-cm portion of soil compared to the 15–30-cm portion. The soil texture at the Napa County (Oakville) vineyard was classified as a loam with approximately 33% sand, 42% silt, and 25% clay and a bulk density value of 1.3 g cm ^-3^. As was observed at the Fresno vineyard, SOC was greater in the upper 0–15-cm portion of the soil in the Napa County vineyard (**Table 2**). While the type of cover crop again had no influence on SOC at either depth, tillage reduced SOC at both depths.

**Table 2:** Soil organic carbon (% by mass), bulk density (g cm^-3^), and total C (Mg ha^-1^) in Fresno County and Napa County.

Factors and treatment	Fresno County (Five points)						Napa County (Oakville)					
	Average SOC (% mass)	*p-value* ^a^	Bulk density (g cm^-3^)	*p*-value	C (t ha^-1^)	*p*-value	Average SOC (% mass)	*p-value*	Bulk density (g cm^-3^)	*p*-value	C (t ha^-1^)	*p*-value
**Tillage system (T)**												
NT	0.70	ns	1.4	ns	14.68	ns	1.44 **a**	**	1.3	ns	28.15 **a**	**
CT	0.69		1.4		14.39		1.37 **b**		1.3		26.62 **b**	
**Cover crop (CC)**												
AG	0.88	ns	1.4	ns	18.56	ns	1.47	ns	1.3	ns	28.67	ns
RV	0.90		1.4		18.93		1.45		1.3		28.33	
PG	0.86		1.4		17.99		1.41		1.3		27.55	
**Depth (D)**												
0–15 cm	0.88 **a**	***	1.4	ns	18.50 **a**	***	1.45 **a**	**	1.3	ns	28.18 **a**	**
15–30 cm	0.50 **b**		1.4		10.57 **b**		1.36 **b**		1.3		26.59 **b**	
**Year (Y)**												
2020	0.64	ns	1.4	ns	13.42	ns	1.30	ns	1.3	ns	25.37	ns
2021	0.69		1.4		14.53		1.40		1.3		27.29	
CC × T	--	ns	--	ns	--	ns	--	ns	--	ns	--	ns
CC × T × D	--	ns	--	ns	--	ns	--	ns	--	ns	--	ns
CC × T × D × Y	--	ns	--	ns	--	ns	--	ns	--	ns	--	ns

^a^ ANOVA was used to compare data (p-value indicated). Letters within columns indicate significant mean separation according to Tukey's honestly significant difference (HSD) test (at p = 0.05), where *: p-value < 0.05; **: p-value < 0.001, and ***: p-value < 0.0001. ^b^ NT, no tillage; CT, conventional tillage; AG, annual grass; RV, residual vegetation; PG, perennial grass; SOC, soil organic carbon; ns, not significant; and --, not applicable.

### 3.2 Soil respiration

**Figure 2:**
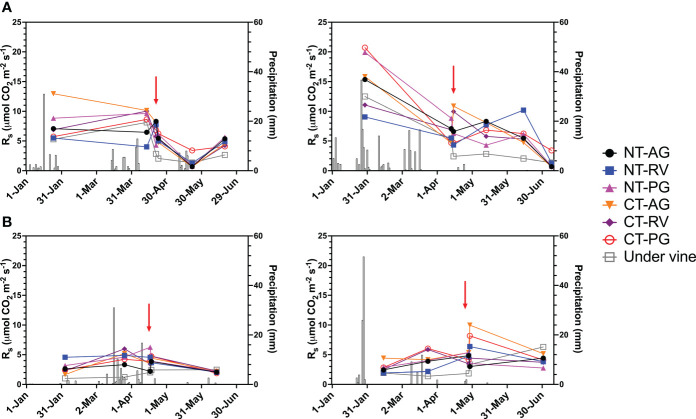
Progression of soil respiration (R_s_) and daily precipitation amounts in two wine grape vineyards, **(A)** Oakville, CA; **(B)** Five Points, CA; 1) 2020; 2) 2021. AG, annual grass; PG, perennial grass; RV, resident vegetation; CT, conventional tillage; NT, no-till. Red arrows indicate tillage for CT treatments.

Across the five readings of R_s_ measured in the interrows at the Fresno County vineyard, when R_s_ readings were averaged to determine the seasonal mean R_s_, no overall differences were observed over the duration of the experiment (**Table 3**). Likewise, the effect of tillage was inconsistent on R_s_ (**Figure 2**). At the Napa County (Oakville) site, tillage also increased R_s_ during the first measurement in the Napa County vineyard. When readings were averaged to yield seasonal R_s_, tillage displayed a stronger overall effect and increased R_s._ There were no interactions observed between the type of cover crop and the tillage system at either site. Finally, under vine, R_s_ was higher in 2021 than that in 2020 at both sites.

**Table 3:** Average soil respiration (R_s_) (t C ha^-1^ year^-1^) analyzed over the 2-year study across five (Fresno) and six (Napa) timepoints as measured in each cover crop and tillage treatment combination and under vine (bare soil).

Fresno County (Five points)								
	Tillage system (T)			Cover crop (CC)				
	NT	CT	*p*-value^a^	AG	RV	PG	Under vine	*p*-value
**2020**								
3-Feb	3.45	2.12	ns	2.15	3.43	2.78	1.03	ns
25-Mar	4.27	5.13	ns	4.26	5.43	4.41	1.23	ns
16-Apr	4.34	3.60	ns	2.85	3.99	5.07	2.04	ns
17-Apr	3.80	4.63	ns	4.14	4.20	4.32	2.42	ns
12-Jun	2.10	2.11	ns	2.12	2.15	2.04	2.46	ns
Season-long Average R_s_	3.59	3.52	ns	3.10	3.84	3.72	1.84	ns
**2021**								
14-Feb	2.41	3.24	ns	3.42 **a**	2.20 **b**	2.86 **b**	1.92 **c**	*
24-Mar	3.35	5.35	ns	4.02 **b**	4.02 **b**	5.01 **a**	1.38 **c**	*
28-Apr	4.99	4.32	ns	5.10	4.12	4.74	1.85	ns
29-Apr	4.29	7.52	ns	6.48	5.39	5.84	3.34	ns
1-Jul	3.66	4.32	ns	4.79	3.76	3.42	6.30	ns
Season-long Average R_s_	3.74	4.95	ns	4.76	3.90	4.37	2.96	ns
CC × T	--	--	ns	--	--	--	--	ns
Year (Y)	--	--	ns	--	--	--	--	ns
CC × T × Y	--	--	ns	--	--	--	--	ns
Napa County (Oakville)								
	Tillage system (T)			Cover crop (CC)				
	NT	CT	*p*-value^a^	AG	RV	PG	Under vine	*p*-value
**2020**								
24 January	7.14 **b**	8.52 **a**	*	10.02**a**	6.19 **b**	7.29 **b**	5.29 **c**	*
13 April	6.70 **b**	9.61 **a**	*	8.30	7.02	9.14	8.13	ns
21 April	6.78	6.90	ns	8.21	6.75	5.56	2.82	ns
23 April	5.52	5.43	ns	5.09	5.15	6.19	2.05	ns
22 May	0.91	1.60	ns	0.67	1.02	2.08	1.36	ns
19 June	5.30	4.27	ns	4.93	4.56	4.86	2.64	ns
Seasonal Average R_s_	5.39 **b**	6.06 **a**	**	6.20	5.12	5.85	3.71	ns
**2021**								
29 January	14.78**b**	15.87**a**	*	15.60 **b**	10.06**c**	20.32**a**	12.48**b**	*
14 April	7.14	5.42	ns	5.89	6.19	6.76	5.29	ns
16 April	5.72	8.50	ns	8.74	7.14	5.45	2.40	ns
14 May	6.78	6.90	ns	8.21	6.75	5.56	2.82	ns
15 June	7.26	5.43	ns	5.09	7.76	6.19	2.05	ns
9 July	0.91	1.60	ns	0.67	1.02	2.08	1.36	ns
Seasonal Average R_s_	7.10 **b**	7.29 **a**	*	7.36	6.49	7.73	4.40	ns
CC × T	--	--	ns	--	--	--	--	ns
Year (Y)	--	--	ns	--	--	--	--	ns
CC × T × Y	--	--	ns	--	--	--	--	ns

^a^ ANOVA was used to compare data (p-value indicated). Letters within columns indicate significant mean separation according to Tukey's honestly significant difference (HSD) test (at p = 0.05), where *: p-value < 0.05; **: p-value < 0.001, and ***: p-value < 0.0001. ^b^ NT, no tillage; CT, conventional tillage; AG, annual grass; RV, residual vegetation; PG, perennial grass; ns, not significant; and --, not applicable.

### 3.3 Grapevine and cover crop NPP

At the Fresno County vineyard, there was an effect of cover crop on pruning wood and leaf C contributions (**Table 4**), whereby the PG reduced C input through the production of annual growth (canes and leaves) compared to the AG and RV. However, this difference did not translate into differences in yield or C input from fruit. Ultimately, no differences in grapevine NPP were observed between different cover crops nor tillage system. Contrary to the Fresno site, at the Napa County (Oakville) vineyard, the annual growth (harvest, pruning wood, leaves) was affected by tillage system rather than cover crop. Grapevines under tillage resulted in higher C contributions from pruning wood and leaves. This contributed to differences in grapevine NPP; whereby tillage increased NPP, RV resulted in the highest NPP compared to AG and then PG. A significant interaction of the two factors was found, and year-to-year differences were also observed at the Napa County site whereby C contributions from harvest and pruning wood were higher in 2021 compared to 2020, thus the same trend was observed among grapevine NPP as well.

**Table 4:** Components of the vineyard’s net primary production (NPP as t ha^-1^ of dry matter), the whole vine’s net carbon balance (NCB), and the vineyard’s net ecosystem carbon balance (NECB) calculated over the 2-year study for six different cover crop and tillage systems.^a,b^.

Treatment		Harvest (Mg C ha^-1^)	Pruning wood (Mg C ha^-1^)	Leaves (Mg C ha^-1^)	Permanent organs (Mg C ha^-1^)	Grapevine NPP (Mg C ha^-1^ year^-1^)	R_s_ Under vine (Mg C ha^-1^ year^-1^)	Grapevine NCB (Mg C year^-1^)	Cover Crop NPP (Mg C ha^-1^)	R_s_ Interrow (Mg C ha^-1^ year^-1^)	SOC (Mg C ha^-1^)	NECB (Mg C ha^-1^ year^-1^)
Tillage (T)	Cover crop (CC)											
**Fresno County (Five points)**												
**2020**												
NT	AG	1.39	1.76	3.02	11.5	17.7	1.84	15.35	1.80	2.81	17.71	23.36
	PG	1.41	1.30	2.97	11.5	17.2	1.84	14.83	1.70	4.02	19.25	22.96
	RV	1.32	1.73	3.53	11.5	18.1	1.84	15.82	2.30	3.94	17.64	23.5
CT	AG	1.25	1.75	3.22	11.5	17.7	1.84	15.54	3.93	3.39	19.81	25.31
	PG	1.48	1.56	3.27	11.5	17.8	1.84	15.39	1.83	3.42	18.97	23.74
	RV	1.65	2.17	2.93	11.5	18.3	1.84	15.66	1.17	3.74	17.64	22.89
**2021**												
NT	AG	1.24	1.61	2.98	11.5	17.3	2.96	14.56	2.00	3.71	17.71	22.24
	PG	1.63	1.25	2.92	11.5	17.3	2.96	14.15	2.15	3.69	19.32	22.69
	RV	1.80	1.53	3.48	11.5	18.3	2.96	14.99	1.83	3.81	17.71	22.54
CT	AG	1.11	1.75	3.18	11.5	17.6	2.96	14.91	4.08	5.81	19.88	23.63
	PG	1.21	1.50	3.09	11.5	17.3	2.96	14.58	2.07	5.06	18.97	22.25
	RV	1.34	2.01	2.88	11.5	17.8	2.96	14.88	1.33	3.98	17.36	21.94
CC		ns	*	*	--	ns	--	ns	**	ns	ns	ns
T		ns	ns	ns		ns		ns	ns	ns	ns	ns
CC × T		ns	ns	ns		ns		ns	ns	ns	ns	ns
Year		ns	ns	ns		ns		*	*	ns	ns	ns
Year × CC		ns	ns	ns		ns		ns	ns	ns	ns	ns
Year × T		ns	ns	ns		ns		ns	ns	ns	ns	ns
Year × CC × T		ns	ns	ns		ns		ns	ns	ns	ns	ns
**Napa County (Oakville)**												
**2020**												
NT	AG	0.15	0.37	0.57	6.7	7.8	3.71	5.71	2.92	5.56	29.25	18.49
	PG	0.17	0.21	0.49	6.7	7.6	3.71	5.47	0.96	5.89	28.28	16.68
	RV	0.14	0.43	0.44	6.7	7.7	3.71	5.63	2.59	4.73	28.76	18.41
CT	AG	0.16	0.51	0.55	6.7	7.9	3.71	5.83	2.96	6.85	29.25	18.00
	PG	0.16	0.32	0.64	6.7	7.8	3.71	5.72	1.19	5.82	27.30	16.61
	RV	0.16	0.77	0.74	6.7	8.4	3.71	6.29	3.20	5.50	27.30	18.29
**2021**												
NT	AG	0.44	0.56	0.61	7.0	8.6	4.40	5.88	3.20	7.25	28.76	17.74
	PG	0.33	0.39	0.53	7.0	8.3	4.40	5.63	1.62	7.70	27.79	16.05
	RV	0.26	0.62	0.47	7.0	8.3	4.40	5.80	3.60	6.68	30.23	18.83
CT	AG	0.37	0.68	0.58	7.0	8.6	4.40	5.97	3.32	7.48	27.30	17.08
PG	0.38	0.48	0.67	7.0	8.5	4.40	5.86	1.47	7.75	26.81	15.72
RV	0.46	0.96	0.77	7.0	9.2	4.40	6.45	3.46	5.64	26.81	18.27
CC		ns	ns	ns	--	*	--	**	**	ns	ns	***
T		ns	**	*		***		***	ns	**	**	*
CC × T		ns	ns	ns		*		*	ns	ns	ns	ns
Year		***	**	ns		***		ns	ns	ns	ns	*
Year × CC		ns	ns	ns		ns		ns	ns	ns	ns	ns
Year × T		ns	ns	ns		ns		ns	ns	ns	ns	ns
Year × CC × T		ns	ns	ns		ns		ns	ns	ns	ns	ns

^a^ ANOVA was used to compare data (p-value indicated). Letters within columns indicate significant mean separation according to Tukey's honestly significant difference (HSD) test (at p = 0.05), where *: p-value < 0.05; **: p-value < 0.001, and ***: p-value < 0.0001. ^b^ NT, no tillage; CT, conventional tillage; AG, annual grass; RV, residual vegetation; PG, perennial grass; NPP; net primary production; NCB, net carbon balance; R_s_, respiration; SOC, soil organic carbon; NECB, net ecosystem carbon balance; ns, not significant; and --: not applicable.

### 3.4 Estimates of net carbon balance at the grapevine and vineyard scale

**Figure 3:**
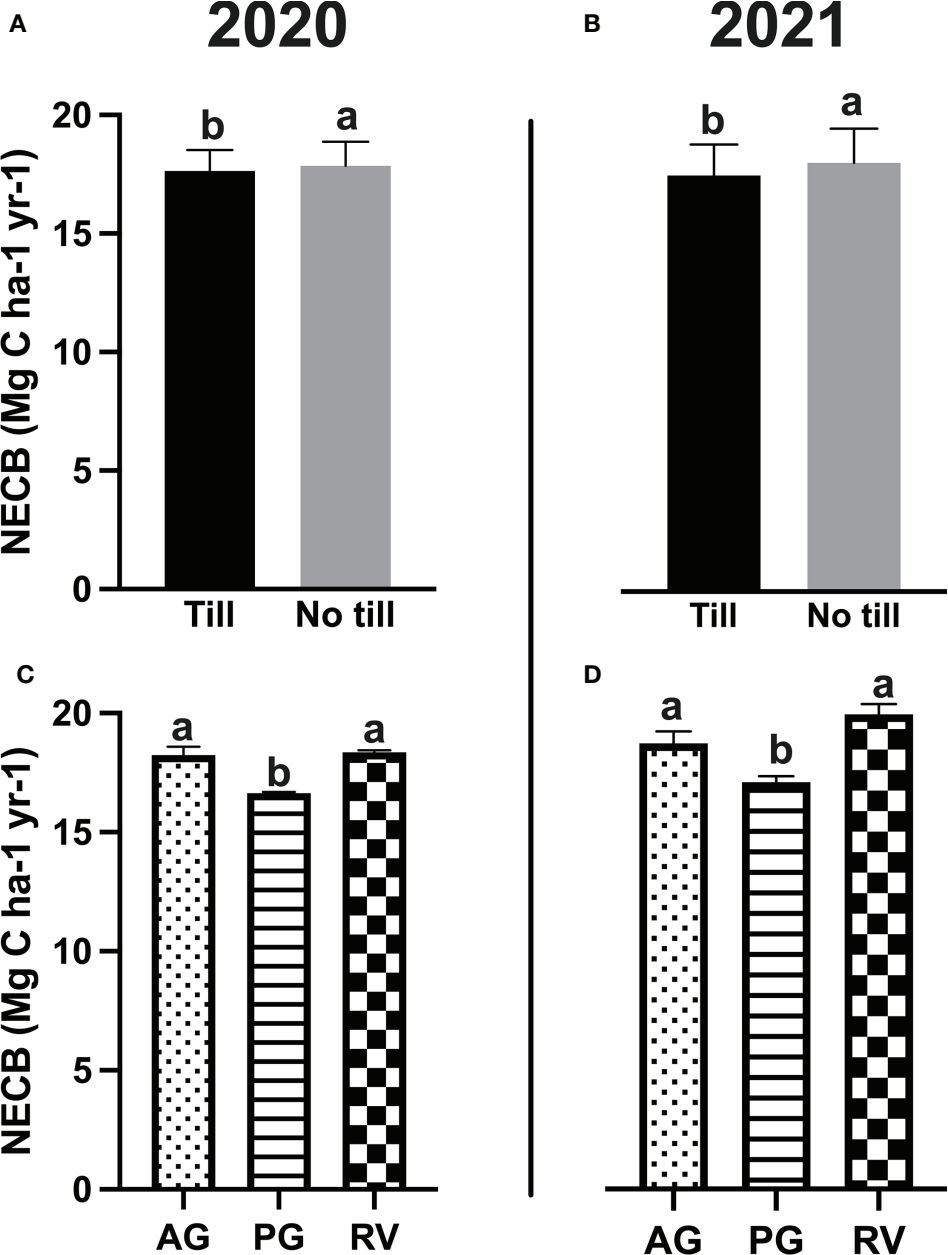
**(A, B)** The net ecosystem carbon balance (NECB) as affected by tillage treatments in a Merlot vineyard in Oakville, CA, USA. **(C, D)** The NECB as affected by cover crop treatment in the same vineyard. Values represent means ± SE. Different letters indicate significant differences (p ≤ 0.05) between respective treatments according to two-way ANOVA followed by Tukey’s honest significant difference (HSD) test. AG, annual grass; PG, perennial grass; RV, resident vegetation.

When harvest mass and R_s_ under vine were subtracted from grapevine NPP to generate the grapevine net carbon balance (NCB), a year-to-year difference was observed at the Fresno County vineyard, as values in 2020 were greater than that of 2021 (**Table 4**). At the Napa County vineyard, there was an effect of cover crop, tillage, and an interaction of the two factors on grapevine NCB. The same pattern as grapevine NPP was observed whereby NCB values were greatest under RV followed by the AG and then PG. Tillage resulted in greater NCB. When SOC adjusted for spatial coverage of the interrow (+), R_s: inter-row_ (-), and NPP_cover crop_ (+) were added to the NCB to yield the NECB for the six different cover crop and tillage systems, no treatment effects were observed at the Fresno County site. At the Napa County site, however, some previous statistical trends from the grapevine NCB reversed when the remaining components of the vineyard ecosystem were added. Overall, PG and tillage reduced the NECB, but there was no interaction between the two factors (**Figure 3**). A greater NECB was also observed in 2020 compared to 2021.

## 4 Discussion

### 4.1 Soil texture plays a key role in the effects of cover crop and tillage on SOC

After 2 years of the adoption of treatments, there were no statistical differences between the type of cover crop on SOC at either experimental vineyard, which agreed with some previous studies when cover crops were implemented and the effects on SOC were monitored only for a short period of time (Belmonte et al., 2018; Novara et al., 2019). However, there were many other studies that have noticed a significant increase in SOM under cover cropping (Morlat and Jacquet, 2003; Steenwerth and Belina, 2008b), and the results might be dependent on the specific climatic, topography, and soil conditions in the experimental sites (Novara et al., 2019; Yu et al., 2019). As previously reported, greater SOC content was generally found in the upper portion of the soil (0–15 cm) compared to the deeper portion (Steenwerth and Belina, 2008b; Mitchell et al., 2017; Yu et al., 2019). Previous literature has shown that this phenomenon was quite consistent among most soil textures of temperate regions (Krull et al., 2001; Tautges et al., 2019), which might be the reason when significant effects were observed, the effectiveness of cover crops on the soil properties might be higher in the upper layer soils than those of deeper layer soils (Mitchell et al., 2017; Tautges et al., 2019). On the other hand, SOC values at both sites were similar with other studies under similar soil texture and climates (Steenwerth et al., 2010; Wolff et al., 2018; Yu et al., 2019). However, the lack of strong effects of tillage on SOC at the Fresno County vineyard can be attributed to the significantly less SOM with more sandy soil texture in the Fresno County vineyard compared to the Napa County vineyard with finer soil texture, which could have possibly led to excessive aeration and permeability in the soil (Yu et al., 2019; Beltrán et al., 2021), hence a very low SOM baseline for the Fresno site. As for the Napa site with CT significantly deducing soil SOC, there has been recent work in the similar Mediterranean climates that is generally in agreement with these observations, reporting increased SOC accumulation under minimum and complete lack of tillage (López‐Bellido et al., 2010; Wolff et al., 2018). However, it should be noted that broad conclusions regarding increased SOC under NT management are nuanced, as other studies have discussed the effects of reduced tillage on SOC content and whether they would be truly beneficial toward plant growth, soil biodiversity, or overall soil health, since SOC has to be decayed or immobilized to be usable by soil microbiomes and plants (Janzen, 2006); and whether the long-term effects in deeper soil layers and the whole soil profile would still accredit the capability of NT on C sequestration (Baker et al., 2007; Blanco-Canqui and Lal, 2008); and whether the conflicting results can be solved by standardizing research methodologies and technologies (Derpsch et al., 2014).

### 4.2 R_s_ and cover crop biomass are main drivers of the NECB

At the Fresno County vineyard, while the soils under cover crop displayed differences in R_s_ in the early season R_s_ measurement events in 2021, ultimately no seasonal differences were observed. This may be due to the higher sand content in the soil at this experimental site (Bouma and Bryla, 2000) and more active cover crops during the spring measurements, causing higher R**_s_** at the early season (Nilahyane et al., 2020). While tillage indeed increased R_s_ values due to oxidation of organic matter from exposed soil aggregates and generally higher soil temperature during the growing season. Overall, these effects were minimal in terms of determining the NECB from the vineyard agroecosystem at the Fresno site.

A similar trend was observed at the Napa County vineyard, where R_s_ interrow showed differences in the early season, despite no significant differences of cover crop type over seasonal average R**_s_** values. CT increased the seasonal average R_s_ values and the annual production, NPP, and NCB. Previous research showed that the higher SOC can be directly linked soil texture, where finer soils showed greater SOC storing capacity than coarser soils (Bird et al., 2003; Zinn et al., 2007; Wiesmeier et al., 2014). On the contrary, there was evidence shown that climate might have a larger effect on SOC than soil texture (Wang et al., 2010). Nevertheless, these two factors might have contributed to the significant difference in R_s_ at the Napa site, where the soil texture was finer and the climate was relatively cooler. However, when the remaining components of the vineyard agroecosystem were added, the NECB was enhanced by NT rather than CT. This indicated that R_s_ and SOC have played a greater role in determining the NECB compared to the other factors, as losses of C through interrow R_s_ and SOC under tillage were large enough to negate the previous increase in NPP. Moreover, the loss of interaction between the two factors, cover crop and tillage, may further confirm that interrow R_s_ plays a significant role in NECB determination and thus the C storage potential of vineyards.

On the other hand, high variability in biomass led to a significant effect of cover crop type on the NECB at both experimental sites. This was likely due to the compensation of C input through biomass from the cover crops implemented in this study, as the AG and RV all showed greater biomasses compared to the low-stature PG. AG and RV have generated greater biomasses compared to low-profile *Poa bulbosa* as the PG as higher biomass-producing plants would generally have higher C-storing capacity in many previous studies (Williams et al., 2011; Agostini et al., 2015; Williams et al., 2020). And eventually, the differences in cover crop biomass capacity led to the observed changes in the NECB, where the greatest values were under RV, followed by AG and PG.

## 5 Conclusion

Our findings provide more evidence of that the vineyard agroecosystem can serve as a C sink for short-term implementation of cover crops with NT practice. Corroborating previous research under sandy soils, tillage and type of cover crop had little to no effect on the NECB. However, under the finer-textured soils, CT reduced the NECB through a reduction in SOC and increase in R_s_, or soil CO_2_ efflux. The type of cover crop also impacted the NECB, as cover crops that produced greater biomass increased the NECB. Ultimately, vineyard site characteristics, including soil texture and climate, were key determinants of the effectiveness of C storage potential, as they can determine SOC and R_s_ of vineyards in Mediterranean vineyard agroecosystems in both Napa and Fresno. Overall, the implementation of NT and cover crop practices should be carefully considered with a thorough understanding of the specific site characteristics to fully maximize their effectiveness.

## Data availability statement

The raw data supporting the conclusions of this article will be made available by the authors, without undue reservation.

## Author contributions

SK designed the trial and acquired the funding. MZ, NT, RY, and LM executed the trial. MZ and NT curated the data. MZ wrote the first version of the manuscript. All authors read and approved the final submitted version of the paper.

## Conflict of interest

The authors declare that the research was conducted in the absence of any commercial or financial relationships that could be construed as a potential conflict of interest.

## Publisher’s note

All claims expressed in this article are solely those of the authors and do not necessarily represent those of their affiliated organizations, or those of the publisher, the editors and the reviewers. Any product that may be evaluated in this article, or claim that may be made by its manufacturer, is not guaranteed or endorsed by the publisher.

## References

[B1] Agostini F. Gregory A. S. Richter G. M. (2015). Carbon sequestration by perennial energy crops: is the jury still out? Bioenergy Res. 8, 1057–1080. doi: 10.1007/s12155-014-9571-0 26855689 PMC4732603

[B2] Alluvione F. Bertora C. Zavattaro L. Grignani C. (2010). Nitrous oxide and carbon dioxide emissions following green manure and compost fertilization in corn. Soil Sci. Soc Am. J. 74, 384–395. doi: 10.2136/sssaj2009.0092

[B3] Alonso F. P. Alvarez E. P. P. Domínguez E. G. (2014). The short term influence of aboveground biomass cover crops on c sequestration and ß glucosidase in a vineyard ground under semiarid conditions. Spanish J. Agric. Res. 12 (4), 1000–1007. doi: 10.5424/sjar/2014124-5818

[B4] Alsina M. M. Fanton-Borges A. C. Smart D. R. (2013). Spatiotemporal variation of event related N2O and CH4 emissions during fertigation in a California almond orchard. Ecosphere 4, 1–21. doi: 10.1890/ES12-00236.1

[B5] Alsina M. M. Smart D. R. Wolff M. W. (2014). Soil carbon sequestration following conservation tillage of a vineyard. AGU Fall Meeting Abstracts 4, B53D–0224.

[B6] Álvaro-Fuentes J. López M. V. Cantero-Martínez C. Arrúe J. L. (2008). Tillage effects on soil organic carbon fractions in Mediterranean dryland agroecosystems. Soil Sci. Soc Am. J. 72, 541–547. doi: 10.2136/sssaj2007.0164

[B7] Baker J. M. Ochsner T. E. Venterea R. T. Griffis T. J. (2007). Tillage and soil carbon sequestration–what do we really know? Agric. Ecosyst. Environ. 118, 1–5. doi: 10.1016/j.agee.2006.05.014

[B8] Belmonte S. A. Luisella C. Stahel R. J. Bonifacio E. Novello V. Zanini E.. (2018). Effect of long-term soil management on the mutual interaction among soil organic matter, microbial activity and aggregate stability in a vineyard. Pedosphere 28, 288–298. doi: 10.1016/S1002-0160(18)60015-3

[B9] Beltrán M. Galantini J. A. Salvagiotti F. Tognetti P. Bacigaluppo S. Sainz Rozas H. R.. (2021). Do soil carbon sequestration and soil fertility increase by including a gramineous cover crop in continuous soybean? Soil Sci. Soc Am. J. 85, 1380–1394. doi: 10.1002/saj2.20257

[B10] Bird M. Kracht O. Derrien D. Zhou Y. (2003). The effect of soil texture and roots on the stable carbon isotope composition of soil organic carbon. Soil Res. 41, 77–94. doi: 10.1071/SR02044

[B11] Blanco-Canqui H. Lal R. (2008). No-tillage and soil-profile carbon sequestration: An on-farm assessment. Soil Sci. Soc Am. J. 72, 693–701. doi: 10.2136/sssaj2007.0233

[B12] Blanco-Canqui H. Shapiro C. A. Wortmann C. S. Drijber R. A. Mamo M. Shaver T. M.. (2013). Soil organic carbon: The value to soil properties. J. Soil Water Conserv. 68, 129A–134A. doi: 10.2489/jswc.68.5.129A

[B13] Borrelli P. Robinson D. A. Fleischer L. R. Lugato E. Ballabio C. Alewell C.. (2017). An assessment of the global impact of 21st century land use change on soil erosion. Nat. Commun. 8, 1–13. doi: 10.1038/s41467-017-02142-7 29222506 PMC5722879

[B14] Bouma T. J. Bryla D. R. (2000). On the assessment of root and soil respiration for soils of different textures: interactions with soil moisture contents and soil CO2 concentrations. Plant Soil 227, 215–221. doi: 10.1023/A:1026502414977

[B15] Brunori E. Farina R. Biasi R. (2016). Sustainable viticulture: The carbon-sink function of the vineyard agro-ecosystem. Agric. Ecosyst. Environ. 223, 10–21. doi: 10.1016/j.agee.2016.02.012

[B16] California Department of Food and Agriculture (CDFA) (2022). California Agricultural statistics review 2020-2021. (Sacramento CA: California Department of Food and Agriculture).

[B17] Carlisle E. Smart D. Williams L. E. Summers M. (2010). California Vineyard greenhouse gas emissions. Sustain. Wine Grow. Available at: org/docs/CSWA%20GHG%20Report_Final.pdf.

[B18] Cataldo E. Salvi L. Sbraci S. Storchi P. Mattii G. B. (2020). Sustainable viticulture: Effects of soil management in vitis vinifera. Agronomy 10, 1949. doi: 10.3390/agronomy10121949

[B19] Cates A. M. Jackson R. D. (2019). Cover crop effects on net ecosystem carbon balance in grain and silage maize. Agron. J. 111, 30–38. doi: 10.2134/agronj2018.01.0045

[B20] Celette F. (2007). Dynamique des fonctionnements hydrique et azoté dans une vigne enherbée sous le climat méditerranéen MS Thesis Fonctionnement et conduite des Systèmes de culture Tropicaux et Méditerranéens Institut national d’études supérieures agronomiques de Montpellier.

[B21] Conant R. T. Easter M. Paustian K. Swan A. Williams S. (2007). Impacts of periodic tillage on soil c stocks: A synthesis. Soil Tillage Res. 95, 1–10. doi: 10.1016/j.still.2006.12.006

[B22] De Bei R. Fuentes S. Gilliham M. Tyerman S. Edwards E. Bianchini N.. (2016). VitiCanopy: A free computer app to estimate canopy vigor and porosity for grapevine. Sensors 16, 585. doi: 10.3390/s16040585 27120600 PMC4851099

[B23] Derpsch R. Franzluebbers A. J. Duiker S. W. Reicosky D. C. Koeller K. Friedrich T.. (2014). Why do we need to standardize no-tillage research? Soil Tillage Res. 137, 16–22. doi: 10.1016/j.still.2013.10.002

[B24] Ferreira C. S. S. Veiga A. Caetano A. Gonzalez-Pelayo O. Karine-Boulet A. Abrantes N.. (2020). Assessment of the impact of distinct vineyard management practices on soil physico-chemical properties. Air Soil Water Res. 13, 1178622120944847. doi: 10.1177/1178622120944847

[B25] Freidenreich A. Dattamudi S. Li Y. C. Jayachandran K. (2021). Soil respiration and carbon balance under cover crop in a no-till tropical fruit orchard. Front. Environ. Sci. 653. doi: 10.3389/fenvs.2021.766638

[B26] Gatti M. Garavani A. Squeri C. Capri C. Diti I. D’Ambrosio R.. (2022). Inter-row floor management is a powerful factor for optimising vine balance in a non-irrigated organic barbera vineyard in northern Italy. Eur. J. Agron. 136, 126490. doi: 10.1016/j.eja.2022.126490

[B27] Gavlak R. G. Horneck D. A. Miller R. O. (1994). Plant, soil, and water reference methods for the western region. Western Rural Dev. Center.

[B28] Janzen H. H. (2006). The soil carbon dilemma: shall we hoard it or use it? Soil Biol. Biochem. 38, 419–424. doi: 10.1016/j.soilbio.2005.10.008

[B29] Jian J. Du X. Reiter M. S. Stewart R. D. (2020). A meta-analysis of global cropland soil carbon changes due to cover cropping. Soil Biol. Biochem. 143, 107735. doi: 10.1016/j.soilbio.2020.107735

[B30] Jobbágy E. G. Jackson R. B. (2000). The vertical distribution of soil organic carbon and its relation to climate and vegetation. Ecol. Appl. 10, 423–436. doi: 10.1890/1051-0761(2000)010[0423:TVDOSO]2.0.CO;2

[B31] Krull E. Baldock J. Skjemstad J. (2001). “Soil texture effects on decomposition and soil carbon storage,” in Net ecosystem exchange CRC workshop proceedings (Citeseer), 103–110.

[B32] Lal R. (2004). Soil carbon sequestration to mitigate climate change. Geoderma 123, 1–22. doi: 10.1016/j.geoderma.2004.01.032

[B33] Lazcano C. Decock C. Wilson S. G. (2020). Defining and managing for healthy vineyard soils, intersections with the concept of terroir. Front. Environ. Sci. 8. doi: 10.3389/fenvs.2020.00068

[B34] López-Bellido R. J. Fontán J. M. López-Bellido F. J. López-Bellido L. (2010). Carbon sequestration by tillage, rotation, and nitrogen fertilization in a Mediterranean vertisol. Agron. J. 102, 310–318. doi: 10.2134/agronj2009.0165

[B35] Maier M. Schack-Kirchner H. Hildebrand E. E. Schindler D. (2011). Soil CO2 efflux vs. soil respiration: Implications for flux models. Agric. For. Meteorol. 151, 1723–1730. doi: 10.1016/j.agrformet.2011.07.006

[B36] Martínez-Lüscher J. Kurtural S. K. (2021). Same season and carry-over effects of source-sink adjustments on grapevine yields and non-structural carbohydrates. Front. Plant Sci. 12. doi: 10.3389/fpls.2021.695319 PMC8350779 34381481

[B37] Mitchell J. P. Shrestha A. Mathesius K. Scow K. M. Southard R. J. Haney R. L.. (2017). Cover cropping and no-tillage improve soil health in an arid irrigated cropping system in california’s San Joaquin valley, USA. Soil Tillage Res. 165, 325–335. doi: 10.1016/j.still.2016.09.001

[B38] Morandé J. A. Stockert C. M. Liles G. C. Williams J. N. Smart D. R. Viers J. H. (2017). From berries to blocks: carbon stock quantification of a California vineyard. Carbon Balance Manage. 12, 1–12. doi: 10.1186/s13021-017-0071-3 PMC5313494 28413849

[B39] Morlat R. Jacquet A. (2003). Grapevine root system and soil characteristics in a vineyard maintained long-term with or without interrow sward. Am. J. Enol. Vitic. 54, 1–7.

[B40] Nilahyane A. Ghimire R. Thapa V. R. Sainju U. M. (2020). Cover crop effects on soil carbon dioxide emissions in a semiarid cropping system. Agrosystems Geosci. Environ. 3, e20012. doi: 10.1002/agg2.20012

[B41] Nistor E. Dobrei A. G. Dobrei A. Camen D. Sala F. Prundeanu H. (2018). N2O, CO2, production, and c sequestration in vineyards: a review. Water Air Soil pollut. 229, 1–10. doi: 10.1007/s11270-018-3942-7

[B42] Novara A. Minacapilli M. Santoro A. Rodrigo-Comino J. Carrubba A. Sarno M.. (2019). Real cover crops contribution to soil organic carbon sequestration in sloping vineyard. Sci. Total Environ. 652, 300–306. doi: 10.1016/j.scitotenv.2018.10.247 30366330

[B43] Panagos P. Borrelli P. Robinson D. (2020). FAO calls for actions to reduce global soil erosion. Mitig. Adapt. Strateg. Glob. Change 25, 789–790. doi: 10.1007/s11027-019-09892-3

[B44] Patiño-Zúñiga L. Ceja-Navarro J. A. Govaerts B. Luna-Guido M. Sayre K. D. Dendooven L. (2009). The effect of different tillage and residue management practices on soil characteristics, inorganic n dynamics and emissions of N2O, CO2 and CH4 in the central highlands of Mexico: a laboratory study. Plant Soil 314, 231–241. doi: 10.1007/s11104-008-9722-1

[B45] Payen F. T. Sykes A. Aitkenhead M. Alexander P. Moran D. MacLeod M. (2021). Soil organic carbon sequestration rates in vineyard agroecosystems under different soil management practices: A meta-analysis. J. Clean Prod. 290, 125736. doi: 10.1016/j.jclepro.2020.125736

[B46] Peregrina F. Larrieta C. Ibáñez S. García-Escudero E. (2010). Labile organic matter, aggregates, and stratification ratios in a semiarid vineyard with cover crops. Soil Sci. Soc Am. J. 74, 2120–2130. doi: 10.2136/sssaj2010.0081

[B47] Porter J. R. Howden M. Smith P. (2017). Considering agriculture in IPCC assessments. Nat. Clim. Change 7, 680–683. doi: 10.1038/nclimate3404

[B48] Powlson D. S. Whitmore A. P. Goulding K. W. T. (2011). Soil carbon sequestration to mitigate climate change: a critical re-examination to identify the true and the false. Eur. J. Soil Sci. 62, 42–55. doi: 10.1111/j.1365-2389.2010.01342.x

[B49] Randerson J. T. Chapin Iii F. S. Harden J. W. Neff J. C. Harmon M. E. (2002). Net ecosystem production: a comprehensive measure of net carbon accumulation by ecosystems. Ecol. Appl. 12, 937–947. doi: 10.1890/1051-0761(2002)012[0937:NEPACM]2.0.CO;2

[B50] Ryan M. G. Law B. E. (2005). Interpreting, measuring, and modeling soil respiration. Biogeochemistry 73, 3–27. doi: 10.1007/s10533-004-5167-7

[B51] Seddaiu G. Porcu G. Ledda L. Roggero P. P. Agnelli A. Corti G. (2013). Soil organic matter content and composition as influenced by soil management in a semi-arid Mediterranean agro-silvo-pastoral system. Agric. Ecosyst. Environ. 167, 1–11. doi: 10.1016/j.agee.2013.01.002

[B52] Šimon T. Javůrek M. Mikanova O. Vach M. (2009). The influence of tillage systems on soil organic matter and soil hydrophobicity. Soil Tillage Res. 105, 44–48. doi: 10.1016/j.still.2009.05.004

[B53] Steenwerth K. Belina K. M. (2008a). Cover crops and cultivation: Impacts on soil n dynamics and microbiological function in a Mediterranean vineyard agroecosystem. Appl. Soil Ecol. 40, 370–380. doi: 10.1016/j.apsoil.2008.06.004

[B54] Steenwerth K. Belina K. M. (2008b). Cover crops enhance soil organic matter, carbon dynamics and microbiological function in a vineyard agroecosystem. Appl. Soil Ecol. 40, 359–369. doi: 10.1016/j.apsoil.2008.06.006

[B55] Steenwerth K. L. Pierce D. L. Carlisle E. A. Spencer R. G. M. Smart D. R. (2010). A vineyard agroecosystem: disturbance and precipitation affect soil respiration under Mediterranean conditions. Soil Sci. Soc Am. J. 74, 231–239. doi: 10.2136/sssaj2008.0346

[B56] Stockmann U. Adams M. A. Crawford J. W. Field D. J. Henakaarchchi N. Jenkins M.. (2013). The knowns, known unknowns and unknowns of sequestration of soil organic carbon. Agric. Ecosyst. Environ. 164, 80–99. doi: 10.1016/j.agee.2012.10.001

[B57] Tautges N. E. Chiartas J. L. Gaudin A. C. M. O’Geen A. T. Herrera I. Scow K. M. (2019). Deep soil inventories reveal that impacts of cover crops and compost on soil carbon sequestration differ in surface and subsurface soils. Glob. Change Biol. 25, 3753–3766. doi: 10.1111/gcb.14762 31301684

[B58] Torres N. Martínez-Lüscher J. Porte E. Yu R. Kaan Kurtural S. (2021). Impacts of leaf removal and shoot thinning on cumulative daily light intensity and thermal time and their cascading effects of grapevine (Vitis vinifera l.) berry and wine chemistry in warm climates. Food Chem. 343. doi: 10.1016/j.foodchem.2020.128447 33131953

[B59] Wang D. D. Shi X. Z. Wang H. J. Weindorf D. C. Yu D. S. Sun W. X.. (2010). Scale effect of climate and soil texture on soil organic carbon in the uplands of northeast China. Pedosphere 20, 525–535. doi: 10.1016/S1002-0160(10)60042-2

[B60] Wiesmeier M. Hübner R. Spörlein P. Geuß U. Hangen E. Reischl A.. (2014). Carbon sequestration potential of soils in southeast Germany derived from stable soil organic carbon saturation. Glob. Change Biol. 20, 653–665. doi: 10.1111/gcb.12384 24038905

[B61] Williams J. N. Hollander A. D. O’Geen A. T. Thrupp L. Hanifin R. Steenwerth K.. (2011). Assessment of carbon in woody plants and soil across a vineyard-woodland landscape. Carbon Balance Manage. 6, 1–14. doi: 10.1186/1750-0680-6-11 PMC3287142 22070870

[B62] Williams J. N. Morandé J. A. Vaghti M. G. Medellín-Azuara J. Viers J. H. (2020). Ecosystem services in vineyard landscapes: a focus on aboveground carbon storage and accumulation. Carbon Balance Manage. 15, 23. doi: 10.1186/s13021-020-00158-z PMC7640672 33141918

[B63] Wolff M. W. Alsina M. M. Stockert C. M. Khalsa S. D. S. Smart D. R. (2018). Minimum tillage of a cover crop lowers net GWP and sequesters soil carbon in a California vineyard. Soil Tillage Res. 175, 244–254. doi: 10.1016/j.still.2017.06.003

[B64] Yu O. T. Greenhut R. F. O’Geen A. T. Mackey B. Horwath W. R. Steenwerth K. L. (2019). Precipitation events, soil type, and vineyard management practices influence soil carbon dynamics in a Mediterranean climate (Lodi, California). Soil Sci. Soc Am. J. 83, 772–779. doi: 10.2136/sssaj2018.09.0345

[B65] Yu R. Kurtural S. K. (2020). Proximal sensing of soil electrical conductivity provides a link to soil-plant water relationships and supports the identification of plant water status zones in vineyards. Front. Plant Sci. 11. doi: 10.3389/fpls.2020.00244 PMC7078246 32218792

[B66] Zhang L. Xue T. Gao F. Wei R. Wang Z. Li H.. (2021). Carbon storage distribution characteristics of vineyard ecosystems in hongsibu, ningxia. Plants 10, 1199. doi: 10.3390/plants10061199 34208416 PMC8231109

[B67] Zinn Y. L. Lal R. Bigham J. M. Resck D. V. S. (2007). Edaphic controls on soil organic carbon retention in the Brazilian cerrado: Texture and mineralogy. Soil Sci. Soc Am. J. 71, 1204–1214. doi: 10.2136/sssaj2006.0014

